# Comparing surgical outcomes of subcutaneous-flap vs. subplatysmal-flap techniques in total open thyroidectomy: a cohort study

**DOI:** 10.1097/MS9.0000000000003040

**Published:** 2025-02-07

**Authors:** Majid Samsami, Behzad Nematihonar, Mohammad Shafiei, Hamed Tahmasbi, Shervin Tavakoli, Seyed Pedram Kouchak Hosseini, Ehsan Adinevand, Alireza Haghbin Toutounchi

**Affiliations:** a Department of Surgery, Imam Hossein Medical and Educational Center, Shahid Beheshti University of Medical Sciences, Tehran, Iran; b Guilan University of Medical Sciences, Rasht, Iran

**Keywords:** flap, head and neck, subcutaneous-flap, subplatysmal-flap, thyroidectomy

## Abstract

**Introduction:**

Various minimally invasive open thyroidectomy techniques have been introduced in surgical centers worldwide, aiming to improve cosmetic outcomes. The conventional open thyroidectomy (CTT) method involves creating a subplatysmal flap through a horizontal incision, which requires cutting the platysma muscle and anterior jugular veins. This study evaluates a modified open total thyroidectomy technique that uses a subcutaneous flap instead of a subplatysmal flap and compares its outcomes with those of the conventional method.

**Methods:**

This observational cohort study included 120 patients from 10 years experience of tertiary medical center. Outcomes were compared between the subcutaneous flap (modified open thyroidectomy) group and the subplatysmal flap (CTT) group.

**Results:**

The modified technique increased the duration of surgery, with the flap type significantly affecting operation time (*P* = 0.016). However, there was no significant difference in hospital stay length between the two groups (*P* = 0.268). The incidence of hematoma and skin ischemia was lower with the subcutaneous flap. No significant differences were observed between the groups in terms of seroma formation, infection, keloid formation, or hypertrophic scars (*P* > 0.05).

**Conclusion:**

The findings suggest that the subcutaneous flap technique is safe and does not make additional risks or complications. Additionally, it appears to offer advantages in specific outcomes.

## Introduction

Thyroidectomy is a surgery that may be performed based on specific conditions and indications^[1]^. While the principles of surgery have remained consistent, advancements in techniques, diagnostic capabilities, understanding of neck anatomy, anesthesia methods, and technology have made thyroidectomy an effective and safe procedure worldwide, with infrequent complications^[2,3]^. Currently, open total thyroidectomy is the most common method used for thyroid surgery, providing exposure to the thyroid lobes and enabling central neck dissection. However, the conventional approach requires a long collar incision (typically 5–8 cm), wide anterior neck flaps, and midline splitting of the strap muscles to expose the thyroid gland, regardless of the extent of thyroidectomy planned^[4-6]^. This often results in a wide neck scar, which affects patient satisfaction^[7-9]^. The desire to improve cosmetic outcomes and reduce complications has led to the introduction of new techniques, including robotic, endoscopic, or non-endoscopic methods through a small neck incision. Various minimally invasive techniques have been used worldwide, aiming to enhance cosmetic outcomes and yield varied therapeutic and cosmetic results^[10,11]^.

**HIGHLIGHTS**

- The primary mission of this study was to evaluate the outcomes of the subplatysmal flap based on 10 years of data, providing evidence of its safety and potential benefits.
- First, there was a concern that the subplatysmal flap could increase the risk of skin ischemia, which this study disproved.
- Second, proponents claimed that the new flap would reduce hematoma due to not severing the anterior jugular veins, a claim confirmed by the study’s findings.
- The final benefit of this new approach is preserving the compartment space and strap muscles of the neck, avoiding entry into other spaces, which can reduce adhesions and preserve the anatomy of the area.

One of the modified techniques involves performing a subcutaneous flap instead of a subplatysmal flap in open thyroidectomy. There is some concern that dissecting the subcutaneous layer may weaken the vascular supply to the flap’s skin, potentially leading to reduced blood flow, ischemia, and necrosis^[12,13]^. This study aims to investigate whether this concern translates to a difference in the incidence of skin ischemia between the two techniques. Additionally, in the conventional method, a transverse incision through the platysma layer warrants cutting the anterior jugular veins located within this layer, which are subsequently ligated. This vascular injury may increase the incidence of postoperative hematoma^[14-16]^. Since the modified technique does not involve cutting the platysmal muscle or damaging these veins, the study also aims to compare the incidence of hematoma between the two groups. Furthermore, the modified method avoids entering the anterior neck muscle space and leaves it untouched. In contrast, the conventional method exposes the muscle space and sheath, which can lead to fibrosis and adhesions in the lateral neck spaces postoperatively. These adhesions can complicate future surgeries such as removing cervical lymph nodes, potentially preventing complete lymph node dissection. Therefore, this study aims to compare the surgical outcomes of the modified technique (modified open thyroidectomy, MTT) versus the conventional open thyroidectomy (CTT).

## Material and methods

### Study design

This is an observational retrospective cohort study conducted at a tertiary medical center. Based on the objectives of this study and similar studies^[14,17]^, the sample size was determined to be approximately 50 patients per group. All the documents of the patients from 2014 to 2024 were investigated and included based on the inclusion and exclusion criteria. Inclusion: Providing informed consent to participate in the study/benign conditions/age of 18–70 years. Exclusion: Intraoperative frozen section/any levels of lymph node dissection/history of head and neck surgery/active inflammatory disease/thyroiditis.

### Technique definition

All patients underwent surgery under general endotracheal anesthesia. In the CTT group, the patient was positioned supine with a slight neck extension. A 3–5 cm collar incision was made one finger-width above the sternal notch. The incision was extended horizontally to the platysma, which was dissected to create a subplatysmal flap, exposing the thyroid. The thyroid vessels and parenchyma were divided using a ligator. The recurrent laryngeal nerve and parathyroid glands were identified and preserved. After hemostasis, a drain was placed in the paratracheal space, and the surgical wound was closed in layers. In the MTT group, the same skin incision was made. However, the dissection was performed subcutaneously without cutting the platysma muscle. The platysma and anterior neck muscles were retracted laterally to expose the thyroid. The rest of the surgery proceeded similarly to the conventional method.

### Data collection

Demographic information included age, gender, and primary pathology. Intraoperative data included the duration of surgery. Postoperative data included the length of hospital stay and surgical complications such as ischemia and skin discoloration, surgical site hematoma, seroma formation, and infection. Follow-up involved assessing pathology results and the development of complications like keloid formation, hypertrophic scars, or other surgical sequelae via telephone follow-ups with patients. The primary outcomes were operation time, length of hospital stay, hematoma, ischemia, seroma formation, and infection. Secondary outcomes were hypertrophic scar and keloid formation.

### Data analysis

Data analysis was conducted using both descriptive and inferential statistics. The research data will be analyzed using IBM SPSS version 27 software. The normality of the data will be assessed using the Kolmogorov–Smirnov test. To compare quantitative variables between the two groups, an independent *t*-test or one-way ANOVA will be used, along with their nonparametric equivalent, the Mann–Whitney *U* test. Fisher’s exact test will be applied to compare categorical variables, and to compare normally distributed quantitative variables within each group (pre- and post-intervention), a *t*-test or its nonparametric equivalent, the Wilcoxon signed-rank test, will be utilized. Regression equations and logistic regression will be employed to explore the relationships between variables. A *P*-value of less than 0.05 will be considered statistically significant for all tests. This article is written in accordance with STROCSS 2021 guidelines^[18]^.

## Results

### Demographics

Reviewing a decade of data from the center, spanning from 2014 to early 2024, it became evident that a total of 107 cases of total thyroidectomy using the modified technique with a subcutaneous flap (MTT) were performed for non-malignant indications, without the need for extensive neck dissection or lymph node dissection. Of these 107 cases, 42 (39.3%) were excluded: 13 due to malignant pathology results, 12 due to age, 7 due to frozen section, 4 due to thyroiditis, and 6 patients who did not consent to participate. Consequently, 65 cases (60.7%) were included in the study. Similarly, cases of thyroidectomy performed over the 10-year period using the subplatysmal flap technique (CTT) were gathered and filtered based on the inclusion criteria, resulting in 55 cases eligible for study entry. The mean follow-up period was 59 months. The earliest included patient’s date of surgery was on March 2014, and the latest one was on December 2023. The minimum follow-up period was 6 months after surgery.

**Figure 1.**
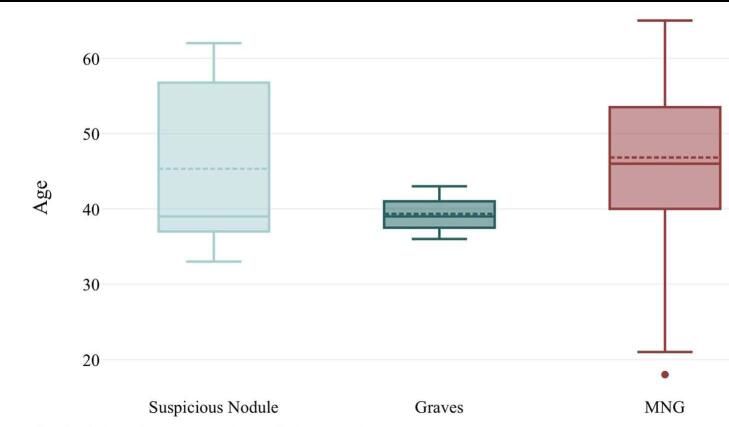
Age means of patients based on preoperative pathology results.

In total, 120 patients were included in the study, with 65 patients (9 males, 56 females) in the MTT group (54.2%) and 55 patients (9 males, 46 females) in the CTT group (45.8%). Of these, 85% were female and 15% were male (*P* < 0.001). While the overall gender difference was statistically significant as an epidemiological finding, there was no significant difference between the two groups in terms of gender distribution (*P* = 0.895). Comparing the two study groups, the mean age was 46.1 years in the MTT group and 47.1 years in the CTT group (*P* = 0.542) (Table 1). Based on preoperative pathology findings, the patients included 111 cases of multinodular goiter (MNG), 6 cases of suspicious nodule (SN), and 3 cases of Graves’ disease (Table 2). MNG constituted 50% of patients in the MTT group and 42.5% in the CTT group. When assessing the mean age of patients by pathology type, MNG patients had the highest average age at 47 years, while Graves’ disease patients had the lowest at 39 years (Fig. 1). However, statistical analysis did not reveal a significant difference in mean age among the pathology types (*P* > 0.05).

**Table 1:** Demographic variables.

Group		MTT	CTT	*P*-value
Age		46.09 ± 9.72	47.10 ± 12.34	0.542
Sex				
Male		9 (%13.8)	9 (16.4%)	0.895
Female		56 (86.2%)	46 (83.6%)

**Table 2:** Distribution of preoperative pathology in two groups.

Group	Pathology	*N* (%)
MTT	MNG	60 (50%)
	SN	2 (1.67%)
	Graves	3 (2.5%)
CTT	MNG	51 (42.5%)
	SN	4 (3.33%)
	Graves	

MNG, multi nodular goiter; SN, suspicious nodule.

### Outcomes

Overall, the average duration of surgery was 143.75 ± 39.14 minutes, with a range from 60 to 270 minutes. Regression analysis revealed a statistically significant difference in the average duration of surgery based on the type of flap used (*P* = 0.005). The average length of hospital stay was 3.5 ± 1.8 days, ranging from 1 to 11 days. However, regression analysis did not show a statistically significant difference in the average length of hospital stay based on the type of flap used (*P* = 0.065) (Table 3).

**Table 3:** Outcomes in the two observed groups.

Methods	Length of stay	Operation time
MTT	3.2 ± 1.36	153 ± 42.44
CTT	3.8 ± 2.2	132.8 ± 31.51
*P*-value	0.268	0.016

**Figure 2.**
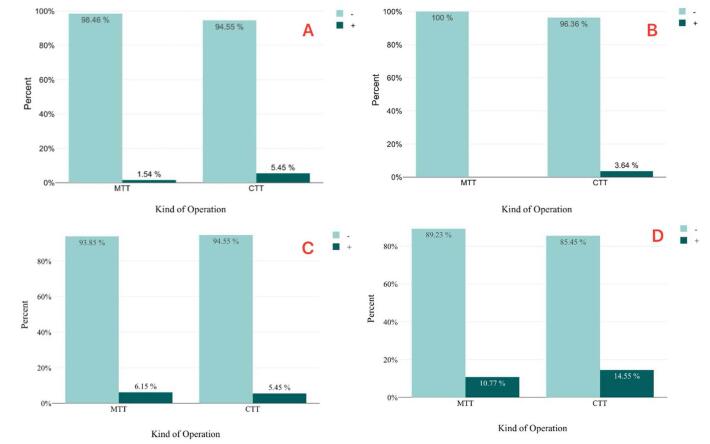
Incidence of complications in two observed groups. (A) Hematoma, (B) ischemia, (C) seroma, and (D) scar formation.

A total of four cases (3.33%) of hematoma were recorded, with one case in the MTT group and three cases in the CTT group. Although the number of hematomas was lower in the MTT group, logistic regression analysis did not show a statistically significant difference in the incidence of hematoma based on the type of flap used (*P* = 0.228). There were two cases (1.66%) of skin ischemia, both occurring in the CTT group, with no cases reported in the MTT group. However, logistic regression analysis did not reveal a statistically significant difference in skin ischemia based on the type of flap used (*P* = 0.075). A total of seven cases (5.83%) required intervention for seroma, including four cases in the MTT group and three cases in the CTT group. Logistic regression analysis did not find a statistically significant difference in seroma formation incidence based on the type of flap used (*P* = 0.870). Finally, 15 individuals (12.5%) developed keloids or hypertrophic scars at the surgical site, with 7 cases from the MTT group and 8 cases from the CTT group. Logistic regression analysis indicated that the type of flap used did not result in a statistically significant difference in the incidence of keloids or hypertrophic scars (*P* = 0.533) (Fig. 2).

## Discussion

Given the lack of consensus on the outcomes and potential benefits of this modification to the conventional open total thyroidectomy, this study compared the two methods regarding the outcomes from creating a subcutaneous flap instead of a subplatysmal flap and avoiding the transverse incision of the platysma muscle based on 10 years of experience with this technique. Kim *et al*^[12]^ initially examined and compared the differences between the two approaches in malignant cases, aiming to introduce this method as a safe and beneficial alternative. Their work laid the foundation for subsequent studies, including the present one, and aligns with our findings. In another study involving 880 patients, Kim *et al*^[13]^ demonstrated that there were no significant differences in complications between the two groups, although the present study indicates fewer complications favoring the subplatysmal flap, marking a step forward. They also reported reduced operation time and hospitalization duration in the intervention group. However, in our study, the operation time increased, and while hospitalization duration was slightly reduced, the difference was not statistically significant. Most other studies have focused on minimally invasive alternatives, such as endoscopic and robotic surgery, which have shown significantly better results in terms of aesthetics and patient satisfaction. Overall, few studies have analyzed the change in flap type for thyroid surgery, making this study one of the few contributions in this area.

The mean overall surgery duration was 143.75 minutes. In the MTT group, the mean surgery duration was 153 minutes, while in the CTT group, it was 132.8 minutes, representing a statistically significant difference (*P* = 0.016). The mean duration of surgery was higher in the MTT group and exceeded the overall mean. Regression and correlation analyses between flap type and surgery duration indicated a significant, positive, and strong association (*P* = 0.005). While this could be seen as a disadvantage for the subcutaneous flap, it is important to consider that the average difference in surgery duration between the two groups is approximately 21 minutes. There are two key points to consider regarding this difference. First, it must be determined whether 21 minutes increase in surgery duration correlates with a significant increase in complications and risks. Studies have shown that differences of less than 30 minutes in surgery duration do not significantly impact the risk of related complications^[17,19]^. Additionally, although efforts were made to enhance the accuracy of surgery duration by excluding cases with pauses for frozen section analysis, the data from operating room records might still be prone to errors. Therefore, the results of this study regarding surgery duration are valuable for comparing the means between the two groups but should not be used as a definitive reference for the duration of an open thyroidectomy. Overall, these findings suggest that this parameter should be evaluated more precisely and standardized in a prospective study. The overall mean hospitalization duration was 3.5 days. In the MTT group, the mean hospitalization duration was 3.2 days, compared to 3.8 days in the CTT group, with no statistically significant difference (*P* = 0.268). Regression analysis confirmed that the type of flap had no significant effect on the length of hospitalization. This result is favorable, as it indicates that the new flap technique did not increase hospitalization duration or associated complications. Additionally, the relationship between surgery duration and length of hospital stay is noteworthy. The lack of an increase in hospitalization duration in the MTT group suggests that, despite the longer surgery time, there were no significant complications leading to prolonged admission.

Regarding skin ischemia, a total of 2 cases (1.66%) were reported among the 120 patients, both occurring in the CTT group. Logistic regression analysis revealed no significant difference between the groups, indicating that the type of flap did not significantly affect the incidence of skin ischemia (*P* = 0.075). This parameter has always been a point of concern, as some argue that creating a skin flap in this area could compromise vascular supply, thereby increasing the risk of ischemia. However, the findings of this study strongly refute this concern, providing crucial evidence for the safety of the new flap technique. The incidence of seroma and infection was also examined. Statistical analysis showed no significant difference between the two groups, indicating that the type of flap did not affect the incidence of seroma (*P* = 0.870). All seroma cases were successfully managed with aspiration, with no additional interventions needed. No cases of surgical site infection were observed among the patients. The seroma rate in this study was 5.8%, consistent with other studies reporting rates up to 7%^[20,21]^. The incidence of infection in this region is rare due to its high vascularity, with reported rates below 0.5% in some studies. This study found no cases of infection^[20]^. Regarding scar formation, 15 cases (2.5%) of hypertrophic scars and keloid formation were reported, aligning with findings from similar studies^[21]^. Statistical analysis showed no significant difference between the groups, indicating that the type of flap did not significantly affect the incidence of keloid or hypertrophic scarring (*P* = 0.533). Multivariate regression analysis also examined the impact of background and independent variables on scar formation, with no significant effects observed (*P* > 0.05). Recent studies suggest that keloid and hypertrophic scars are influenced more by the location of the incision and factors such as ethnicity and darker skin tones rather than the length or type of incision^[21,22]^.

Limitations of the present study are the retrospective design, variability in surgeon experience, and the single-center setting. A very restrictive inclusion/exclusion criteria were necessary for reducing the bias and standardizing both methods to compare, but it may limit the generalizability of the results to other conditions such as malignant cases. This foundational work provides a basis for future clinical trials to further studies and validate these outcomes with greater precision. However, the significant advancements in endoscopic and robotic surgery, particularly for head and neck procedures including thyroidectomy, raise questions about the future relevance of open thyroidectomy.

## Conclusion

This study demonstrates the safety and potential benefits of the modified flap technique. The findings indicate that the subplatysmal flap does not increase the risk of skin ischemia, may reduce the incidence of hematoma by avoiding damage to the anterior jugular veins, and preserves the compartment space and strap muscles of the neck by avoiding unnecessary dissection into other areas.

## Data Availability

The datasets generated during and/or analyzed during the current study are available from the corresponding author upon request.

## Research registration unique identifying number (UIN)

Research Register, researchregistry10537.

## References

[1] Haugen BR Alexander EK Bible KC. 2015 American Thyroid Association management guidelines for adult patients with thyroid nodules and differentiated thyroid cancer: the American Thyroid Association guidelines task force on thyroid nodules and differentiated thyroid cancer. Thyroid 2016;26:1–133. 26462967 10.1089/thy.2015.0020 PMC4739132

[2] Biello A Kinberg EC Wirtz EDT. StatPearls. Treasure Island (FL): StatPearls Publishing; 2023.

[3] Yilmaz H Akkus C Damburaci N. Sonoelastographic evaluation of recurrent thyroid nodules in patients with operated recurrent nodular goiters. Ultrasound Med Biol 2022;48:209–16. 34782167 10.1016/j.ultrasmedbio.2021.10.008

[4] Woo J Kim H Kwon H. Impact of multifocality on the recurrence of papillary thyroid carcinoma. J Clin Med 2021;10:5144. 34768664 10.3390/jcm10215144 PMC8584384

[5] Robenshtok E Neeman B Reches L. Adverse histological features of differentiated thyroid cancer are commonly found in autopsy studies: implications for treatment guidelines. Thyroid 2022;32:37–45. 34779278 10.1089/thy.2021.0268

[6] Knudsen N Perrild H Christiansen E. Thyroid structure and size and two-year follow-up of solitary cold thyroid nodules in an unselected population with borderline iodine deficiency. Eur J Endocrinol 2000;142:224–30. 10700715 10.1530/eje.0.1420224

[7] Welbourn RB. Highlights from endocrine surgical history. World J Surg 1996;20:603–12. 8661638 10.1007/s002689900093

[8] Lee MC Park H Lee BC. Comparison of quality of life between open and endoscopic thyroidectomy for papillary thyroid cancer. Head Neck 2016;38:E827–31. 25917054 10.1002/hed.24108

[9] Park CS Chung WY Chang HS. Minimally invasive open thyroidectomy. Surg Today 2001;31:665–69. 11510599 10.1007/s005950170066

[10] Gagner M. Endoscopic subtotal parathyroidectomy in patients with primary hyperparathyroidism. Br J Surg 1996;83:875. 8696772 10.1002/bjs.1800830656

[11] Dralle H Machens A Thanh PN. Minimally invasive compared with conventional thyroidectomy for nodular goitre. Best Pract Res Clin Endocrinol Metab 2014;28:589–99. 25047208 10.1016/j.beem.2013.12.002

[12] Kim K Kang SW Kim JK. Surgical outcomes of minimally invasive thyroidectomy in thyroid cancer: comparison with conventional open thyroidectomy. Gland Surg 2020;9:1172–81. 33224792 10.21037/gs-20-512 PMC7667120

[13] Kim SY Kim HJ Chang H. Modified version of minimally invasive open thyroidectomy using an unilateral incision. Asian J Surg 2021;44:1166–71. 33814255 10.1016/j.asjsur.2021.02.024

[14] Chong KH Wu MH Lai CW. Comparison of surgical outcome between conventional open thyroidectomy and endoscopic thyroidectomy through axillo-breast approach. Ci Ji Yi Xue Za Zhi 2020;32:286–90. 10.4103/tcmj.tcmj_109_19 PMC7485670 32955515

[15] Chen C Huang S Huang A. Total endoscopic thyroidectomy versus conventional open thyroidectomy in thyroid cancer: a systematic review and meta-analysis. Ther Clin Risk Manag 2018;14:2349–61. 30584310 10.2147/TCRM.S183612 PMC6287425

[16] Govednik CM Snyder SK Quinn CE. Minimally invasive, nonendoscopic thyroidectomy: a cosmetic alternative to robotic-assisted thyroidectomy. Surgery 2014;156:1030–37. 25104462 10.1016/j.surg.2014.06.056

[17] El-Labban GM. Minimally invasive video-assisted thyroidectomy versus conventional thyroidectomy: a single-blinded, randomized controlled clinical trial. J Minim Access Surg 2009;5:97–102. 20407568 10.4103/0972-9941.59307 PMC2843132

[18] Mathew G Agha R. the STROCSS Group. STROCSS 2021: strengthening the reporting of cohort, cross-sectional and case-control studies in surgery. Int J Surg 2021;96:106165. 34774726 10.1016/j.ijsu.2021.106165

[19] Riedinger CB Fantus RJ Matulewicz RS. The impact of surgical duration on complications after transurethral resection of the prostate: an analysis of NSQIP data. Prostate Cancer Prostatic Dis 2019;22:303–08. 30385836 10.1038/s41391-018-0104-3

[20] Ramouz A Rasihashemi SZ Daghigh F. Predisposing factors for seroma formation in patients undergoing thyroidectomy: cross-sectional study. Ann Med Surg 2017;23:8–12. 10.1016/j.amsu.2017.09.001 PMC5612789 28970942

[21] Kim JH Sung JY Kim YH. Risk factors for hypertrophic scar. Wound Repair Regen 2012;20:304–10. 22530655 10.1111/j.1524-475X.2012.00784.x

[22] Arora A Swords C Garas G. The perception of scar cosmesis following thyroid and parathyroid surgery: a prospective cohort study. Int J Surg 2016;25:38–43. 26602967 10.1016/j.ijsu.2015.11.021

